# Enhanced Fc and complement activity of Fc‐modified avelumab boosts anti‐tumor activity but promotes NK cell fratricide

**DOI:** 10.1002/cti2.70098

**Published:** 2026-05-05

**Authors:** Lachlan J Dobson, Barry D Hock, Ariana HT Drabble, Liping Goddard, Judith L McKenzie, Sean A MacPherson, Alexander D McLellan

**Affiliations:** ^1^ Department of Microbiology and Immunology University of Otago Dunedin New Zealand; ^2^ Haematology Research Group, Department of Pathology and Biomedical Science University of Otago Christchurch New Zealand; ^3^ Haematology Department Christchurch Hospital Christchurch New Zealand; ^4^ Department of Histology, Faculty of Medicine University of Indonesia Jakarta Indonesia

**Keywords:** antibody‐dependent cellular cytotoxicity, complement‐dependent cellular cytotoxicity, immune checkpoint inhibitor, monoclonal antibodies

## Abstract

**Objectives:**

The anti‐PD‐L1 antibody avelumab has demonstrated efficacy across multiple cancer types. Avelumab primarily blocks the PD‐1/L1 immune checkpoint, while inducing antibody‐dependent cellular cytotoxicity (ADCC) from CD16a^+^ NK cells. However, subsets of patients possess lower‐affinity CD16a allotypes that limit ADCC capacity of monoclonal antibodies. In this study, we modified the Fc domain of avelumab with characterised mutations to enhance ADCC across all CD16a allotypes.

**Methods:**

Comparisons between the wild‐type avelumab and modified ‘AveFc5M’ were carried out to assess the impacts of introduced mutations on PD‐L1 blockade, ADCC induction and complement‐dependent cytotoxicity (CDC) induction. To assess ADCC, a range of PD‐L1^+^ target cells were coated with mab prior to incubation with effector cells, with both high‐ and low‐affinity CD16a allotypes represented. Human serum was employed for assessing CDC.

**Results:**

Both antibodies exhibited equivalent PD‐L1 blocking activity. Notably, the modified AveFc5M displayed significantly enhanced ADCC across diverse tumor cell–effector cell combinations. Fc mutations also conferred the ability to mediate complement‐dependent cytotoxicity (CDC) against a PD‐L1^+^ lymphoma cell line. However, short term activation of NK cells or long‐term expansion on artificial antigen presenting cells, led to upregulation of PD‐L1 on NK cells. Addition of avelumab or AveFc5M to these NK cell populations induced a marked reduction in viable NK cells via ADCC‐mediated fratricide.

**Conclusion:**

These findings demonstrate that Fc engineering of avelumab can substantially augment ADCC and CDC activity against PD‐L1^+^ tumors. However, the enhanced effector function of AveFc5M led to NK cell fratricide, potentially limiting NK cell‐dominated anti‐cancer responses.

## Introduction

Blockade of the PD‐1/PD‐L1 checkpoint axis with monoclonal antibodies induces durable therapeutic responses, but only in a subset of patients.^1^ In addition to checkpoint blockade, certain monoclonal antibodies (mAb) can modulate anti‐tumor activity by signalling via their Fc regions.^2^ Engineering such mAb to either reduce or enhance Fc‐mediated activity therefore has the potential to improve their efficacy.^3^

The PD‐L1 mAb, avelumab, is effective in multiple cancer types.^4^ While avelumab primarily blocks the PD‐1/PD‐L1 axis, its intact IgG_1_ Fc region enables binding from FcR^+^ cells and permits anti‐tumor responses, such as antibody‐dependent cellular cytotoxicity (ADCC) from CD16a^+^ NK cells.^5^, ^6^ The dual action of avelumab likely contributes to its clinical efficacy particularly in the setting of strongly PD‐L1^+^ tumors. Through glyco‐engineering of Fc residues, CD16a‐binding can be enhanced, reportedly increasing anti‐tumor activity.^7^ This increase was further enhanced by blockade of the inhibitory FcR, CD32b. Both a pre‐clinical study and clinical trial data suggest that increasing avelumab's Fc functions correlated with improved efficacy.^7^, ^8^

Multiple Fc mutations have been identified that, in combination, can enhance CD16a activity while decreasing affinity for CD32b.^9^ Margetuximab was generated by incorporating these Fc modifications into the HER2 mAb, trastuzumab.^9^ Studies to date indicate that margetuximab outperforms trastuzumab, especially in inducing ADCC from NK cells with a low‐affinity CD16a allotype (158F/F).^9^, ^10^ In addition, the changes in FcR engagement increase IFN‐γ release by effector cells; subsequently driving increased PD‐L1 expression on associated tumor cells.^11^

The Fc modifications incorporated into margetuximab demonstrate a viable strategy for enhancing the Fc‐mediated effector functions of other therapeutic antibodies, including avelumab. In this study, we introduced defined Fc mutations into avelumab and evaluated their effects on PD‐L1 binding, as well as their capacity to induce antibody‐dependent cellular cytotoxicity (ADCC) and complement‐dependent cytotoxicity (CDC). We also highlight the presence of PD‐L1 on NK cells and discuss the implications this has for the development and use of ADCC‐enabled anti‐PD‐L1 mAb.

## Results

### Fc modifications retain PD‐L1 binding and blockade

**Figure 1:**
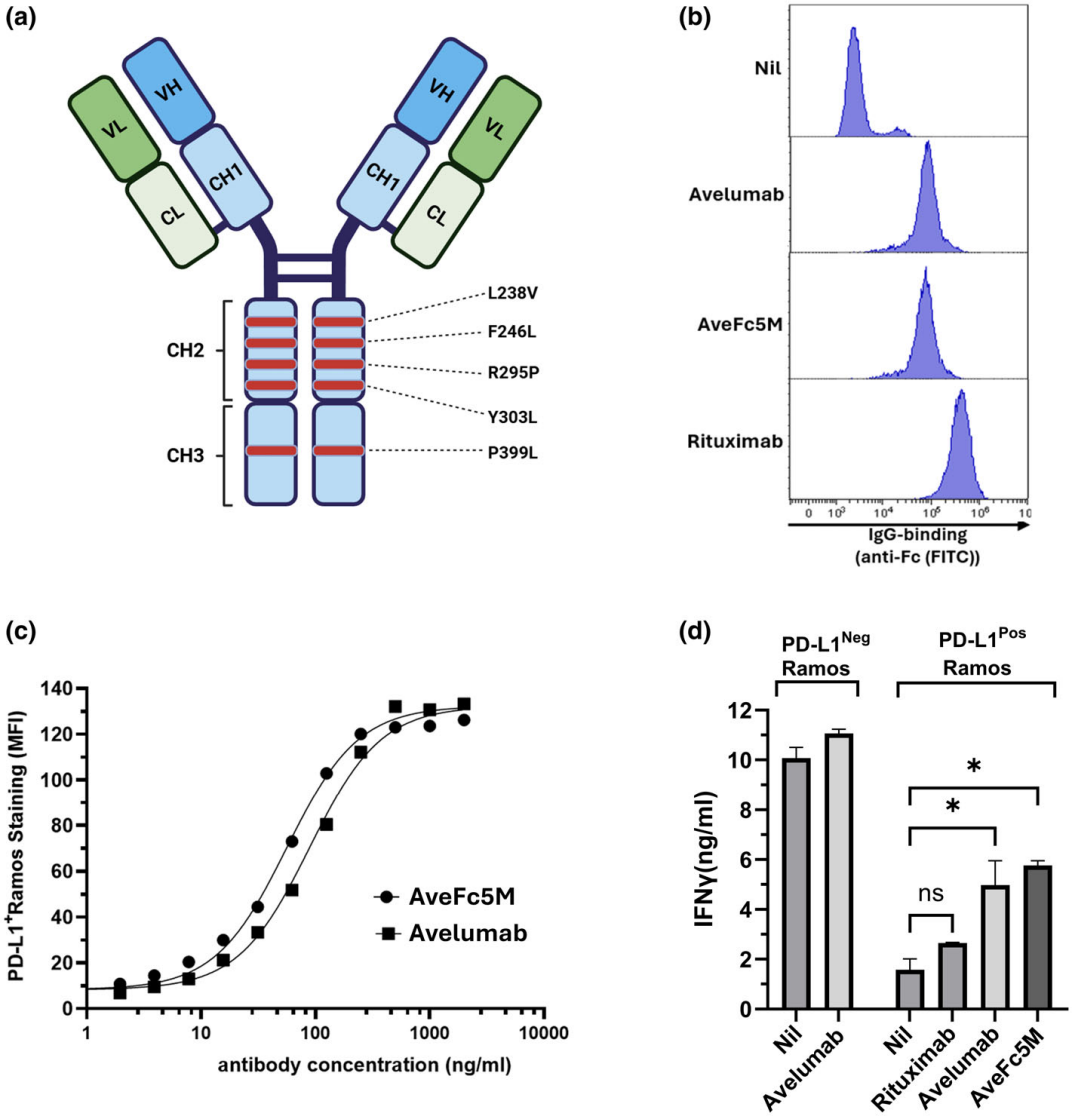
Antibody binding and blockade activity. **(a)** Illustration of Fc mutations present in AveFc5M. **(b, c)** The binding of avelumab and AveFc5M to PD‐L1^+^ Ramos was analysed by flow cytometry following staining with FITC‐anti‐Fc. **(b)** Representative histogram of staining observed using the indicated mAb. **(c)** Dose dependence of mAb binding shown as four‐parameter dose–response curves of staining intensity (MFI) versus antibody concentration. Data are from a representative experiment of three performed. **(d)** Ability of mAb to reverse PD‐L1^+^ Ramos induced suppression of IFNϒ release. PHA‐stimulated CD16^neg^ peripheral blood mononuclear cell (PBMC) were co‐cultured (72 h) with wild‐type or PD‐L1^+^ Ramos in combination with the indicated mAb. Supernatant levels of IFN‐γ were determined by ELISA, and data are from a representative experiment (*n* = 2) shown as histograms (mean ± SEM). Bars and asterisks indicate significance of differences between the indicated treatment pairs following analysis by ANOVA in combination with Holm–Sidak multiple comparisons.

AveFc5M is a Fc‐modified variant of wild‐type avelumab with five amino acid substitutions in the Fc region (Figure 1a). Modifying the heavy chain retained binding kinetics to PD‐L1^+^ Ramos cells compared with wild‐type avelumab. Both mAb had similar binding curves and the ratio of EC_50_ values was not significantly different (*P* = 0.248) from 1 (*n* = 3, mean ratio = 1.2, 95% CI = 0.54–1.99).

The ability of AveFc5M and avelumab to block the functional effects of PD‐1/PD‐L1 interaction was analysed in an IFN‐_ϒ_ release assay. Both mAb specifically increased IFN‐_ϒ_ release induced in co‐cultures of CD16^Neg^ PBMC responders and suppressor PD‐L1^+^ lymphoma cells (Figure 1d). While the CD20‐targeting rituximab bound strongly to Ramos, no blocking of the PD‐1:PD‐L1 axis was detected.

### Fc modifications enhance ADCC of tumor cell lines

**Figure 2:**
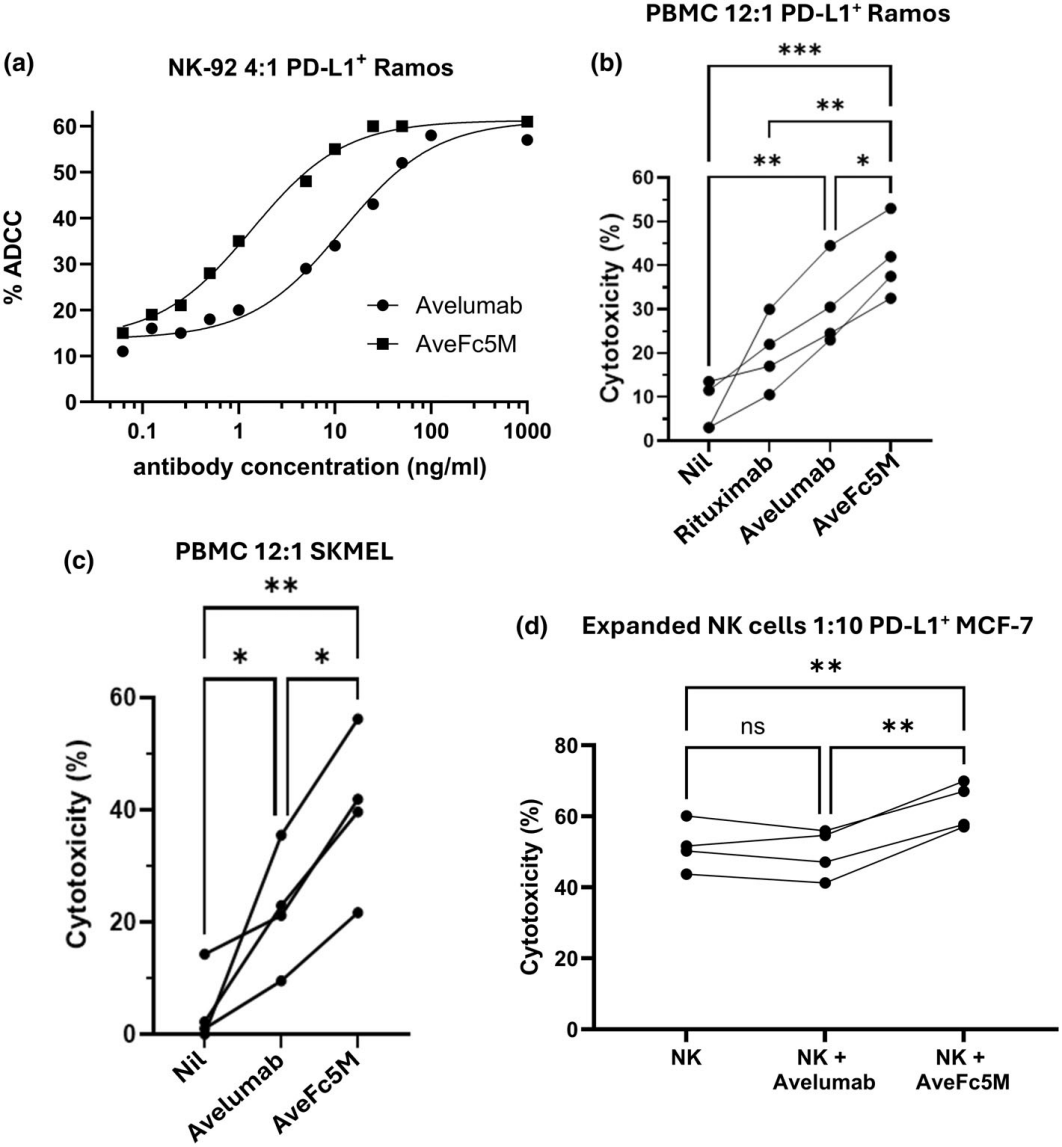
Antibody‐dependent cellular cytotoxicity (ADCC) activity of mAb. The ability of avelumab and AveFc5M to induce ADCC was analysed. ADCC was performed using combinations of effectors and targets cultured in the presence or absence of indicated mAb prior to analysis of target viability. **(a)** CD16‐NK‐92 effectors expressing high‐affinity CD16a (V/V) targeting lymphoma cells (PD‐L1^+^ Ramos) at effector:target (E:T) ratio = 4:1. Data are shown as a four‐parameter dose–response plot and are from a representative experiment of three performed. **(b)** Peripheral blood mononuclear cell (PBMC) effectors plus PD‐L1^+^ Ramos at E:T ratio = 12:1. **(c)** IL‐2 activated PBMC plus melanoma targets (SK‐MEL) at E:T ratio = 12:1. **(d)** Expanded NK cells from donors expressing low‐affinity CD16A (F/F) plus breast cancer cells (PD‐L1 + MCF7) at E:T = 1:10. **(b–d)** Data from four separate experiments are shown as linked points and asterisks indicate significant differences following ANOVA in combination with Holm–Sidak multiple comparisons.

Avelumab and AveFc5M were assessed for ADCC induction against PD‐L1^+^ tumor cell lines (Figure 2). Respective EC50 values were first determined using PD‐L1^+^ Ramos as the target and the NK92 cell line as effectors. NK92 cells express a single high‐affinity (158 V/V) allotype of CD16a and lack PD‐L1 expression (Figure 2a and Supplementary figure 1). Avelumab and AveFc5M had similar maximal levels of cytotoxicity, though the introduced mutations provided AveFc5M with a distinct shift in its activity curve and reduction of EC50. The ratio of EC50 values (avelumab/AveFc5M) had a mean value = 9 (95% CI = 8.16–10.07, *n* = 3) and was significantly different from one (*P* < 0.0001).

Avelumab and AveFc5M were again compared, but with PBMC as effectors at a low effector:target (E:T) ratio (Figure 2b). Due to strong CD20 expression on Ramos, the performance of avelumab and AveFc5M was compared with rituximab, a known inducer of ADCC. Consistent with previous reports, rituximab induced low but significant levels of ADCC at these E:T ratios.^12^ Despite lower expression of PD‐L1 than CD20 (Figure 1b), avelumab and AveFc5M induced higher ADCC than rituximab, with AveFc5M significantly outperforming wild‐type avelumab.

Avelumab and AveFc5M were further analysed for their ability to induce ADCC of an IFN‐_ϒ_‐activated melanoma cell line, SKMEL (Figure 2c). These cells express relatively low levels of PD‐L1 compared with PD‐L1^+^ Ramos (Supplementary figure 2). PBMC activated with IL‐2 were applied as effectors (E:T 12:1). Once again, both avelumab and AveFc5M induced significant increases in ADCC, but levels induced by AveFc5M were significantly higher than those observed with avelumab.

Further analysis was performed using NK cells that were expanded from the PBMC of donors with the low‐affinity F/F allele of CD16a (Figure 2d and Supplementary figure 3). The expanded NK cells were then used as effectors in an assay using a PD‐L1^+^ breast cancer cell line (MCF‐7) as the target. In contrast to the other ADCC systems, high levels of background cytotoxicity were observed in the absence of antibody. Although avelumab did not significantly modulate cytotoxicity, a significant increase was observed in the presence of AveFc5M.

### Fc modifications enable CDC activity

**Figure 3:**
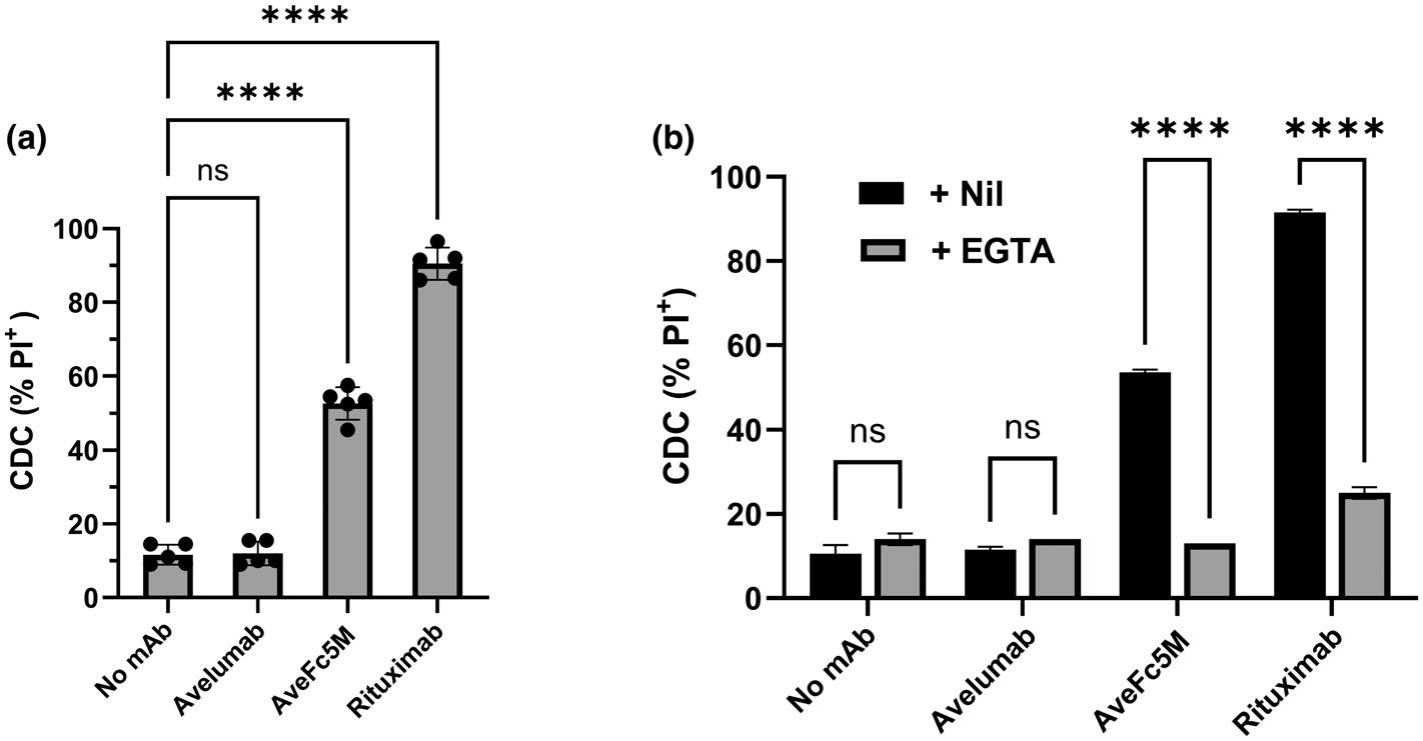
Ability of mAb to induce complement‐dependent cytotoxicity (CDC) of target cells. PD‐L1^+^ Ramos was incubated with nil, rituximab, avelumab or AveFc5M then exposed to human complement prior to determination of CDC using propidium iodide (PI) staining in combination with flow cytometry. **(a)** CDC induced in the presence of indicated mAb. Data from five individual experiments are shown as histograms and asterisks indicate significant differences between groups following ANOVA in combination with Holm–Sidak multiple comparisons. **(b)** CDC observed following exposure to complement supplemented with nil or EGTA to inhibit the alternative pathway. Data from a representative experiment of two performed are shown as histograms (mean ± SEM) and asterisks indicate significance following comparison of nil versus EGTA using paired *t*‐test in combination with *Bonferroni* correction for multiple comparisons.

The ability of avelumab and AveFc5M to induce CDC was analysed with PD‐L1^+^ Ramos as a target and human serum as a complement source (Figure 3). The addition of avelumab did not significantly increase the low levels of cell death observed in the presence of control antibody (Figure 3a). However, the addition of AveFc5M resulted in a significant increase in CDC. As PD‐L1^+^ Ramos expresses higher levels of CD20 than PD‐L1 (Figure 1b), rituximab was utilised as a positive control and induced > 90% cell death. The CDC induced by both AveFc5M and rituximab was inhibited by EGTA/Mg^2+^, confirming that CDC proceeds via the classical pathway with no contribution from the alternative pathway (Figure 3b).

### Fc modifications enhance ADCC of activated NK cells

NK cells can upregulate PD‐L1 following stimulation with target cells and/or cytokines.^11^, ^13^, ^14^, ^15^ Therefore, introducing Fc‐enhancing modifications to avelumab could amplify ADCC‐mediated fratricide in which the NK cells act as both the effectors and targets.

**Figure 4:**
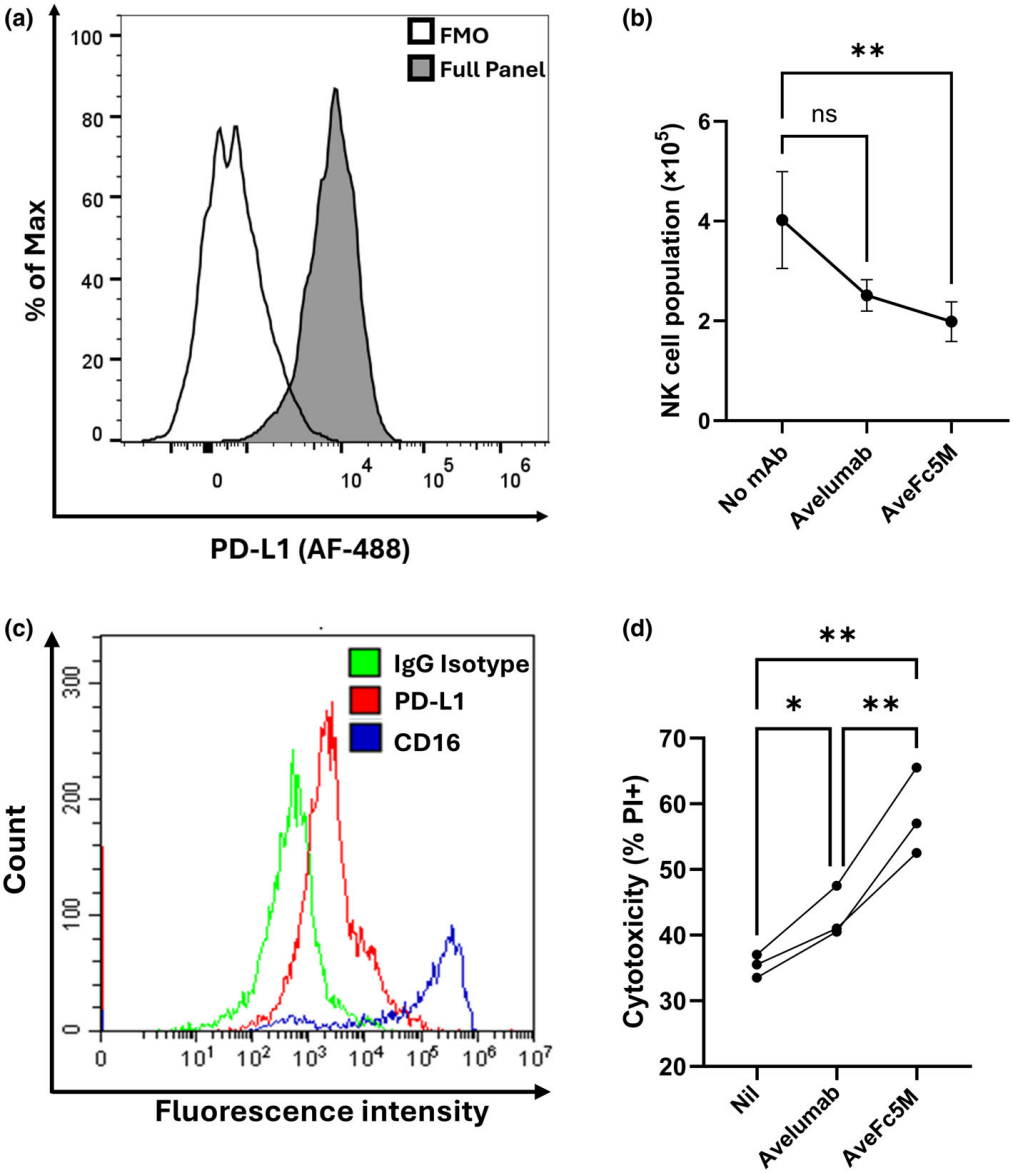
Ability of mAb to induce NK fratricide. In these assays, NK cells were both the effectors and targets. NK cells used for analysis were either **(a, b)** expanded by 12‐day culture of peripheral blood mononuclear cell (PBMC) with aAPC or **(c, d)** purified from PBMC then activated 16 h with K562 + IL‐2. **(a, c)** NK cell expression of PD‐L1 and CD16 was analysed by flow cytometry and representative data shown as histograms. **(b, d)** mAb‐induced fratricide was analysed by incubating NK cells with either nil, avelumab or AveFc5M prior to determination of viability **(b)**. Expanded NK were cultured 16 h with mAb prior to enumeration. Data from eight individual donors are shown as mean ± SEM with significant differences between indicated groups following Two‐way ANOVA in combination with Bonferroni multiple comparisons. **(d)** Activated NK cells were cultured for 4 h with mAb prior to determination of cytotoxicity using propidium iodide (PI) staining in combination with flow cytometry. Data from four separate experiments are shown as linked points and asterisks indicate significant differences between indicated groups following ANOVA in combination with Holm–Sidak multiple comparisons.

Expanded NK cell populations were generated from PBMC using a 12‐day IL‐2^+^K562‐based feeder cell stimulation. Indeed, expanded NK populations uniformly expressed PD‐L1 (Figure 4a). While the addition of avelumab did not show significant changes, AveFc5M resulted in a substantive decrease in viable NK cell numbers (Figure 4b).

Activated NK cells were also prepared by 16 h stimulation with K562 + IL‐2. All cells expressed low levels of PD‐L1 and the majority (55–86%) expressed CD16 (Figure 4c). While avelumab induced NK cell death, AveFc5M significantly enhanced this observation, further increasing NK cell fratricide (Figure 4d).

## Discussion

Fc modifications have the potential to improve the efficacy of mAb, including immune checkpoint inhibitors.^3^ Avelumab has induced responses in numerous cancer types, and modulating its impact on Fc receptors may further boost these effects.^4^, ^7^, ^8^ Therefore, the *in vitro* impact of Fc mutations on avelumab binding and function was analysed.

The Fc mutations introduced into avelumab were the same mutations utilised to derive margetuximab, the Fc engineered variant of the HER2 antibody, trastuzumab. These mutations conferred margetuximab with both enhanced binding to CD16 and reduced binding to inhibitory CD32b. Consequently, antigen affinity is preserved while ADCC activity is enhanced—particularly in the setting of low target antigen density and/or effectors carrying the low‐affinity CD16a allele.

In the current study, we demonstrate a similar effect of these mutations on avelumab. AveFc5M had the same PD‐L1‐binding and blocking activity as wild‐type avelumab. However, the ADCC capabilities of AveFc5M were considerably enhanced relative to avelumab in a range of tumor–effector combinations. When targeting high density antigen with high‐affinity CD16a effectors, AveFc5M displayed a nine‐fold decrease in EC50 versus avelumab; a similar observation was made when using margetuximab versus trastuzumab.^9^ Likewise, AveFc5M induced significantly higher levels of ADCC against melanoma targets with comparatively low PD‐L1 expression.

Expanded NK populations are the focus of considerable research as potential cellular immunotherapies.^16^ In the current study, NK cells were expanded from donors with homogeneous expression of the low‐affinity CD16a allele. When used as effectors, these cells had moderate levels of natural cytotoxicity against a PD‐L1^+^ breast cancer line. Cytotoxicity via ADCC was significantly increased by addition of AveFc5M but not avelumab. These results underline the potential for AveFc5M to increase anti‐tumor cytotoxicity in a range of tumor–effector settings.

It has been reported that NK cells can express PD‐L1 following exposure to targets and/or cytokines.^11^, ^13^, ^14^, ^15^ The presence of Fc‐competent PD‐L1 antibodies in this context could therefore initiate fratricide of the NK cells. Analysis of both expanded NK cells and activated NK cells demonstrated that both avelumab and AveFc5M could induce fratricide; however, AveFc5M induced significantly more fratricide than wild‐type avelumab in activated NK cells expressing lower levels of PD‐L1. NK cell fratricide resulting from therapeutic antibodies has been previously described primarily in the setting of CD38 antibodies used to treat myeloma.^17^ Similar to PD‐L1, the immune checkpoint, TIGIT, is upregulated on activated NK cells. Recent studies have also demonstrated that Fc‐competent TIGIT antibodies can induce NK cell fratricide, and this is thought to contribute to the unexpectedly low clinical efficacy of these antibodies.^18^ Our results suggest that the impact of NK cell fratricide should also be considered in the context of Fc‐competent PD‐L1 antibodies.

The Fc mutations utilised in the current study were identified using screening assays that analysed their impact on Fc affinity for FcR.^19^ Their potential impact on CDC activity was either not investigated or reported to have a neutral or inhibitory impact. The ability of an antibody to induce CDC is influenced by multiple factors, including antigen density and clustering, distance of the epitope from the cell surface and the ability of bound antibodies to form hexamers.^20^ Hexamer formation is influenced by the structure of the Fc region and can be altered by mutations within the Fc.^19^ However, the impact of such mutations on CDC will be dependent on both the spatial characteristics of the particular antigen/antibody combination and the target cell's expression of complement regulatory proteins. The Fc mutations introduced into margetuximab were reported to increase C1q binding in addition to their impact on FcR affinity.^9^ However, it was not reported whether this increased binding could result in CDC. Our findings demonstrate that in the context of CDC‐sensitive target cells, the Fc mutations provide AveFc5M with the capacity to induce CDC. The induction of complement activation results not only in direct cell lysis but also generates opsonins and anaphylatoxins that can modulate the type and magnitude of immune responses.^21^

The overall impact of Fc‐mediated effects on the efficacy of PD‐L1 antibodies is unclear. Blocking the PD‐1/PD‐L1 interaction provides the primary rationale for the use of these antibodies. Avelumab, in contrast to other therapeutic PD‐L1 mAb, can engage FcR, and it has been reported this results in not only increased ADCC but also enhanced NK cell and dendritic cell activation.^5^ Consequently, further enhancement of Fc/FcR engagement could increase therapeutic efficacy. Our finding that modifying Fc residues can significantly increase ADCC in multiple tumor/effector settings and also induce CDC in some settings appears to support that approach. However, the finding that fratricide of NK cells is also increased in the presence of Fc‐variant AveFc5M represents a hurdle to potential application in a therapeutic setting, particularly where NK cells might play a major role in anti‐tumor responses. Such settings might include NK cell‐sensitive tumors that have evaded T‐cell responses or following the adoptive transfer of expanded NK cells or CAR NK cells. Although NK‐92 cells typically show minimal PD‐L1 expression under standard culture conditions, they may upregulate PD‐L1 after infusion *in vivo*, which could similarly render them vulnerable to fratricide.

In this study, we show that AveFc5M induces fratricide in both expanded NK cells and activated NK cells that express PD‐L1. In contrast, unmodified avelumab failed to trigger appreciable fratricide in expanded NK cells and elicited substantially less fratricide in activated NK cells than AveFc5M. These differences reflect the dependence of ADCC on the affinity between the antibody Fc region and CD16 on NK cells. This is not only affected by the presence/absence of affinity‐enhancing mutations, but also by the allotype of the CD16 receptors. The Fc mutations in this study have a strong enhancing effect in settings where the NK cells bear the low‐affinity allotype. The magnitude of this effect is reduced in donors carrying the high‐affinity CD16 allotype,^9^ and under conditions of excess antibody, wild‐type avelumab and AveFc5M elicit similar levels of target‐cell lysis. Thus, the reduced fratricide observed with avelumab in this study likely reflects the lower‐affinity CD16 allotypes present in the donor NK cells. These findings underscore the critical role of CD16 allotype in determining the magnitude of ADCC.

Further analyses will be required to determine whether PD‐L1 antibodies with either native or enhanced Fc activity add or detract from the beneficial effects of PD‐L1 blockade. Overall, this study shows that although Fc engineering can markedly enhance the cytotoxic activity of avelumab, this creates a double‐edged effect: the antibody can drive fratricide of PD‐L1^+^ NK cells.

## Methods

### Antibodies and flow cytometry

Stocks of therapeutic mAb were obtained from injection vials of rituximab (MabThera, Roche), and pembrolizumab (Keytruda, Merck). Both wild‐type avelumab and AveFc5M were generated in‐house as previously described by simultaneous transfection of heavy and light chain sequences into ExpiCHO‐S cells.^22^ Binding of human antibody to target cells was detected by labelling with FITC conjugated anti‐human Fc. PD‐L1‐FITC and PD‐1‐PE were obtained from BD Biosciences. Peripheral blood mononuclear cell (PBMC) and NK cells were stained with AF700‐anti‐CD3, BV421‐anti‐CD56 and BV711‐anti‐CD16, all obtained from BioLegend. Flow cytometry data were acquired with Beckman Coulter Cytoflex S and Cytek Aurora flow cytometers and analysed with the FlowJo v10.10.0 software.

### Cell culture and generation of cell lines

The R10 media used in cell‐based experiments was RPMI (Sigma, St Louis, MO) supplemented with 10% heat inactivated foetal calf serum (FCS, Invitrogen, Auckland, New Zealand) glutamine and penicillin/streptomycin unless otherwise indicated.

The B‐cell line, Ramos, was obtained from ATCC (Manassas, VA, USA) and maintained in R10 media. PD‐1^+^ Ramos and PD‐L1^+^ Ramos were generated as previously described using cDNA sequences cloned into Sleeping Beauty vectors under the control of the EF‐1α promoter.^22^, ^23^ A natural killer cell line (GFP‐CD16‐NK‐92) that had been transduced to express GFP and high‐affinity CD16 (V/V) was obtained from the ATCC and maintained in supplemented R10 media as described.^22^, ^24^ CD16 expression by GFP‐CD16‐NK92 was routinely monitored and remained high throughout culture (Supplementary figure 1).

K562‐derived artificial antigen presenting cells (aAPC) expressing membrane‐bound IL‐15, IL‐21, CD86 and 4‐1BBL were generated as described previously^25^ and maintained in R10. The luciferase‐expressing MCF‐7 (HTB‐22) cell line was maintained in Dulbecco's Modified Eagle Medium (DMEM) (Gibco) supplemented with 10% FCS and 1% penicillin/streptomycin. To generate PD‐L1^hi^ cells, MCF‐7 cells were transfected with a 5:1 ratio of pSBbi PD‐L1 plasmid and CMV CAT transposase with lipofectamine 3000 (Invitrogen L3000015; Thermo Fisher) and selected with 2 μg mL^−1^ puromycin.

Peripheral blood mononuclear cells were isolated from the blood of healthy, consenting donors through layering on Histopaque‐1077 (Sigma‐Aldrich 10771; Merck, Darmstadt, Germany) and centrifugation. In a number of experiments, CD16^Neg^ PBMC were prepared by immunomagnetic negative selection using CD16 mAb (HO80) in combination with anti‐mouse IgG microbeads and LD Columns according to the manufacturers' (Miltenyi Biotec) instructions.

To expand NK cells, PBMC were exposed to K562 aAPC at a 1:1 ratio and cultured in R10 supplemented with 50 U mL^−1^ recombinant IL‐2 (Peprotech 200‐02; Gibco). Half of the media was replenished every second day, with full replenishment of IL‐2. NK cells were expanded for 14 days, with routine passages to encourage expansion and maintain viability.

### CD16a genotyping

During PBMC isolations, gDNA was isolated from samples of whole blood with a Monarch gDNA Isolation Kit (New England Biolabs T3010S; Ipswich MA, USA). gDNA samples were subjected to PCR with primers flanking the CD16a‐encoding sequences. The resulting PCR product was sanger sequenced and mapped to a wild‐type reference genome with the Geneious Prime software.

CD16a Fwd primer: ATGGCAAAGGCAGGAAGTAT

CD16a Rev primer: CAACTTCCCAGTGTGATTGC

### Complement‐dependent cytotoxicity assay

Complement‐dependent cytotoxicity (CDC) was analysed using a flow cytometry‐based assay as described previously.^22^ In brief, target cells were incubated (20 min) with mAb (20 μg mL^−1^) and then, following the addition of human serum (10% final) as a complement source, incubated (2 h/37°C) prior to the addition of propidium iodide (PI) and determination of percent PI^+^ non‐viable cells by flow cytometry. In specificity experiments, the serum was supplemented with 10 mm EGTA as indicated.

### ADCC assays

#### Ramos

Prior to use, the target‐cell Ramos was labelled with Cell Trace Yellow (CTY, Invitrogen) as previously described.^22^ For PBMC‐mediated ADCC, CTY^+^ targets (20 μL, 6 × 10^6^ mL^−1^) were incubated (30 min RT^−1^) in a 96‐well V bottom plate with 10 μL mAb (20 μg mL^−1^) prior to the addition of PBMC (20 μL, 7.5 × 107 mL^−1^). Following incubation (2 h, 37°C) and addition of sytoxBlue (1 μm, Thermos Fischer scientific), the proportion of sytoxblue^+^ targets was analysed by flow cytometry. For NK‐92‐mediated ADCC, CTY^+^ targets (40 μL, 1.25 × 10^6^ mL^−1^) were incubated (30 min RT^−1^) with 40 μL mAb (2000–0.125 ng mL^−1^) prior to the addition of NK92 (40 μL, 5 × 10^6^ mL^−1^), further incubation (4 h, 37°C) and addition of SytoxBlue as above.

#### SK‐MEL‐28

Prior to use SK‐MEL‐28, targets were incubated overnight with IFN_ϒ_ (100 ng mL^−1^) to induce PD‐L1 expression then harvested using accutase (Gibco) and fluorescently labelled by incubation (30 min, 37°C) with 10 nm Calcein AM in 20% AIM V media/HBSS. Activated PBMC effectors (act‐PBMC) were prepared by culture of PBMC (5 × 10^6^ mL^−1^, 16 h) with IL‐2 (100 U mL^−1^). ADCC using SK‐MEL‐28/act‐PBMC was performed as described above for Ramos/PBMC. Cytotoxicity was calculated based on the number of viable targets (bright Calcein AM^+^ Sytoxblue^Neg^ cells) detected by flow cytometry following acquisition of a fixed volume.^26^

#### PD‐L1^hi^ MCF‐7‐Luc

Targets (5 × 10^4^ well^−1^) were seeded into 96‐well plates then incubated (1 h at 37°C) with 10 μg mL^−1^ mAb prior to media replacement with expanded NK cells at a 1:10 effector to target ratio. Following 24 h, co‐cultures were incubated with the firefly luciferase one‐step kit (Pierce 16 197; Thermo Fisher) and bioluminescence was measured with a Varioskan Lux (Thermo Fisher VL0000D0) as previously reported.^25^ Cytotoxicity was calculated by comparing the bioluminescence measured from co‐cultures with that of target cells only.

#### NK cell fratricide ADCC assay

In these assays, the NK cells were both the targets and effectors. (1) NK cells were expanded from PBMC for 12 days with K562 aAPC. NK cell purity was confirmed through flow cytometry (CD3^−^, CD56^+^), with all expanded populations being over 87% purity. NK cells were seeded at 1 × 10^6^ cells mL^−1^ prior to 24 h incubation with 10 μg mL^−1^ mAb. Following incubation, cells were enumerated with trypan blue staining and a Luna ii cell counter (Logo Biosystems; Annandale, VA, USA; L40002) and compared with a no‐mAb control. (2) Activated NK cells were prepared by 24 h culture of purified NK cells (3 × 10^6^ cells mL^−1^) in the presence of 100 U mL^−1^ IL‐2 and K562 (3 × 10^5^ cells mL^−1^). Prior to addition, K562 were pre‐labelled with the fluorescent lipophilic membrane stain DiO. Activated NK cells were seeded at 4 × 10^6^ cells mL^−1^ (25 μL) and incubated for 20 min with 10 μg mL^−1^ mAb at room temperature prior to the addition of 100 μL media and further incubated for 3 h at 37°C. Following the addition of PI, NK cell viability was determined by flow cytometry using DiO fluorescence to discriminate NK cells from K562.

### PD‐L1 blockade assay

CD16^Neg^ PBMC were utilised as responders and added (1.5 × 10^5^ well^−1^) to a 96‐well plate in combination with PD‐L1^+^ Ramos (7.5 × 10^4^ well^−1^), antibodies (20 μg mL^−1^) and PHA (1 μg mL^−1^). Following 72 h culture, supernatants were collected and IFN_ϒ_ levels determined by sandwich ELISA (R&D systems).

## Author contributions

**Lachlan J Dobson:** Conceptualization; investigation; methodology; writing – original draft; validation; writing – review and editing; software; formal analysis; data curation. **Alexander D McLellan:** Conceptualization; funding acquisition; writing – review and editing; writing – original draft; resources; supervision. **Liping Goddard:** Investigation; data curation. **Judith L McKenzie:** Investigation; data curation. **Ariana HT Drabble:** Investigation; data curation. **Sean A MacPherson:** Conceptualization. **Barry D Hock:** Conceptualization; investigation; writing – original draft; writing – review and editing; validation; methodology; software; formal analysis; data curation; supervision.

## Conflict of interest

The authors declare no conflict of interest.

## Ethic statement

This study involved isolating immune cells from the blood of consenting, healthy donors and was approved by the University of Otago Ethics Committee (ethics approval number: H18/089).

## Supporting information


Supplementary figure 1–3

## Data Availability

All data relevant to the study are included in the article and accompanying Supplementary material.
