# Supplementary material for: Enhanced Fc and complement activity of Fc‐modified avelumab boosts anti‐tumor activity but promotes NK cell fratricide
Source: Clin Transl Immunology. 2026 May 5;15(5):e70098. doi: 10.1002/cti2.70098 (PMC13144752; doi:10.1002/cti2.70098)
Supplement: Supplementary file 1 — Supplementary figure 1–3 [file CTI2-15-e70098-s001.docx]

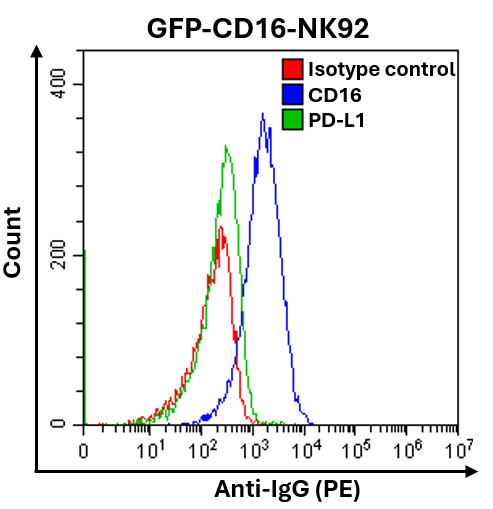


**Supplementary figure 1.** Long-term expression of CD16 and PD-L1 on GFP-CD16-NK92 cell line. GFP-CD16-NK92 cells were cultured over 6 months, with 3 freeze-thaw cycles prior to staining for CD16 and PD-L1. Antigens were stained with primary (IgG) antibodies and subsequently detected with anti-IgG (PE).


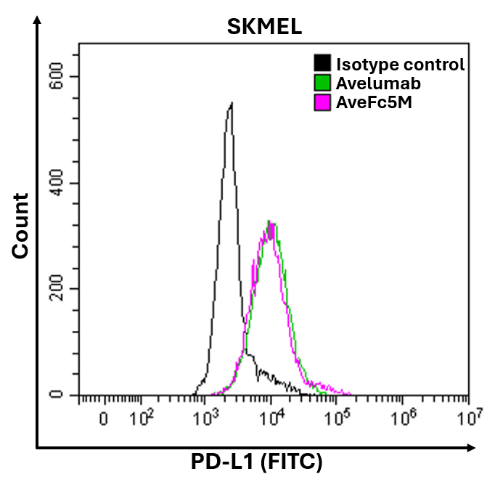

**Supplementary figure 2.** PD-L1 expression on SKMEL cell line. SKMEL cells were exposed to IFN-y and subsequently stained for PD-L1 with either Avelumab or AveFc5M as primary antibodies. Detection was then carried out with anti-IgG (FITC).


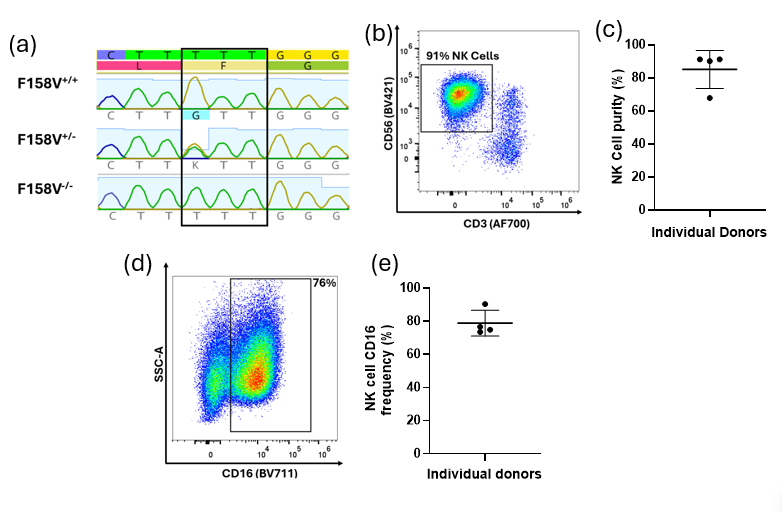


**Supplementary Figure 3.** Healthy donors were screened for CD16 allotypes through PCR and sanger-sequencing of gDNA (a). Pure NK cells were expanded from donors homozygous for low-affinity (F158) CD16. Resulting populations were highly enriched for NK cells (b,c) with high frequencies of CD16^+^ NK cells (d,e).
